# Effectiveness of group reminiscence for improving wellbeing of institutionalized elderly adults: study protocol for a randomized controlled trial

**DOI:** 10.1186/1745-6215-15-408

**Published:** 2014-10-25

**Authors:** Andrea Gaggioli, Chiara Scaratti, Luca Morganti, Marco Stramba-Badiale, Monica Agostoni, Chiara AM Spatola, Enrico Molinari, Pietro Cipresso, Giuseppe Riva

**Affiliations:** Applied Technology for Neuro-Psychology Laboratory, Institute for Treatment and Research (I.R.C.C.S.) Istituto Auxologico Italiano, Via Magnasco 2, 20149 Milan, Italy; Department of Geriatrics and Cardiovascular Medicine, Institute for Treatment and Research (I.R.C.C.S.) Istituto Auxologico Italiano, Via Mosè Bianchi 90, 20149 Milan, Italy; Nursing Home Monsignor Bicchierai, Institute for Treatment and Research (I.R.C.C.S.) Istituto Auxologico Italiano, Via Mosè Bianchi 90, 20149 Milan, Italy; Psychology Research Laboratory, San Giuseppe Hospital, Institute for Treatment and Research (I.R.C.C.S.) Istituto Auxologico Italiano, Via Cadorna 90, 28824 Verbania, Italy; Department of Psychology, Catholic University of Sacred Heart, Largo A. Gemelli 1, 20123 Milan, Italy

**Keywords:** Reminiscence, Group Activities, Group Reminiscence, Older Adults

## Abstract

**Background:**

Group reminiscence therapy is a brief and structured intervention in which participants share personal past events with peers. This approach has been shown to be promising for improving wellbeing and reducing depressive symptoms among institutionalized older adults. However, despite the considerable interest in reminiscence group therapy, controlled studies to determine its specific benefits as compared to generic social interactions with peers (group conversations about everyday subjects) are still lacking.

**Methods/Design:**

We have designed a randomized controlled trial aimed at comparing the effects of group reminiscence therapy with those of group recreational activity on the psychological wellbeing of an institutionalized sample of older adults. The study includes two groups of 20 hospitalized elderly participants: the experimental group and the control group. Participants included in the experimental group will receive six sessions of group reminiscence therapy, while the control group will participate in a recreational group discussion. A repeated-measures design will be used post-intervention and three months post-intervention to evaluate changes in self-reported outcome measures of depressive symptoms, self-esteem, life satisfaction, and quality of life from baseline.

**Discussion:**

The protocol of a study aimed at examining the specific effects of group reminiscence therapy on psychological wellbeing, depression, and quality of life among institutionalized elderly people is described. It is expected that the outcomes of this trial will contribute to our knowledge about the process of group reminiscence, evaluate its effectiveness in improving psychological wellbeing of institutionalized individuals, and identify the best conditions for optimizing this approach.

**Trial registration:**

This trial was registered with ClinicalTrials.gov (registration number: NCT02077153) on 31 January 2014.

## Background

It has been estimated that in Europe the elderly population (aged over 65) will increase significantly over the next few decades, with the number of seniors reaching over 150 million by 2060. An even more dramatic growth will be experienced in the over-eighties population, which will triple to more than 60 million individuals by the same year [1].

As a consequence of this demographic shift, the percentage of dependent seniors in need of institutional care in nursing homes will increase proportionally. A major clinical and policy challenge posed by residential care is the high prevalence of mental health problems among institutionalized elderly individuals. Therefore, the development and provision of effective mental health care interventions for nursing home residents has become an important goal for researchers and practitioners [2].

### Reminiscence therapy

Reminiscence therapy has been proposed as a potentially effective strategy to improve quality of life and psychological wellbeing for elderly nursing home residents [3]. This approach involves the recollection, review, and re-evaluation of personally experienced past events. It is believed that reminiscence therapy can help elderly individuals by increasing self-acceptance, providing perspective, and enabling the resolution of past conflicts [4, 5].Three main reminiscence modalities are identified in literature: simple reminiscence, life review, and life-review therapy [6, 7]. The first approach works best with older adults who have relatively good mental health and involves the simple recollection of positive autobiographical events, with the goal of fostering positive emotions. In contrast, life review focuses on both positive and negative memories, encompassing the entire life span. The goal of this intervention is to perform a critical analysis of one’s life history and achieve ego integrity [8]. Finally, life-review therapy is aimed specifically at elderly persons suffering from mental conditions, with particular applicability to patients with a diagnosis of depression. By assisting patients to reframe negative memories in a positive way, life-review therapy focuses on restructuring beliefs and on building self-efficacy and coping skills. However, it should be noted that the definition of these modalities is often merged [7]. The structure and format of reminiscence interventions also vary considerably across studies. Generally, reminiscence treatments last six weeks or longer, and include at least one or two sessions per week, each session lasting between one and two hours. Moreover, reminiscence activities can be performed either one-to-one or in groups. Reminiscence groups include between six and eight participants and are usually managed by institutional care workers or psychologists.

The effectiveness of reminiscence therapy has been investigated by several meta-analyses [6, 9–13]. Pinquart *et al*. [13] analyzed 57 controlled studies, of which eight compared reminiscence with a control condition. Reminiscence was associated with a large effect size (d =1.00), comparable with cognitive behavioral therapy (CBT). These findings lead the authors to conclude that reminiscence and CBT are effective modalities of treatment for depression in older adults. A more recent review by Pinquart and Forstmeier [6] aggregated results of 128 selected studies. Findings showed that reminiscence interventions produced small to moderate improvements of depressive symptoms (g =0.57) and of other indicators of mental health, such as ego integrity (g =0.50), purpose in life (g =0.73), and death preparation (g =0.52). In addition, it was found that improvements of depression were greater than improvements of positive mental health and cognitive performance. With respect to moderator variables, results of this meta-analysis showed that life-review therapy had stronger effects on depression than either life review or simple reminiscence. However, this effect was explained by higher levels of depression symptoms in life-review patients at pre-test. There was no significant difference between the one-to-one and group formats. In addition, the number of sessions had no moderating effects.

In summary, the findings of previous meta-analytic studies indicate that reminiscence therapy is a promising non-pharmacological approach to improve mental health in elderly individuals. However, more randomized controlled trials are needed to assess the effectiveness of this strategy and its specific benefits for institutionalized seniors [14].

## Methods/Design

### Aim of the study

Based on the need for further randomized controlled trials in this area, the present study aims at assessing the effects of group reminiscence on psychosocial wellbeing in institutionalized, non-demented elderly individuals. First, we will examine the specific effects of simple reminiscence as compared to a placebo intervention in which participants are engaged in everyday conversations (discussion of current events). The findings of previous meta-analyses are inconclusive on this issue. The review carried out by Bohlmeijer *et al*. [9] found no significant differences between studies that used an active placebo and those that used a wait-list condition. In contrast, the Pinquart and Forstmeier meta-analysis [6] observed larger improvements in positive wellbeing if the control condition received no intervention as compared to standard conversation, indicating that socialization activities can also bring about positive emotional states.

Second, we intend to assess the impact of reminiscence on a broad range of psychosocial outcome indicators, including depression symptoms, positive wellbeing (self-esteem, life satisfaction), and quality of life. Since we aim to evaluate the specific effectiveness of simple reminiscence by comparing it to conversation on current events, clinically depressed residents will not be included in the trial. More specifically, we will examine four hypothesis. Hypothesis one is that we expect an improvement of self-esteem, evaluated through the Rosenberg Self-Esteem Scale [15], in the experimental group by the end of the protocol when compared to the control group. Hypothesis two is that we expect an increase of life satisfaction, evaluated through the Life Satisfaction Index [16], in the experimental group by the end of the protocol when compared to the control group. Hypothesis three is that we expect an increase of the perceived quality of life, evaluated through the WHOQOL-Old Scale [17], in the experimental group by the end of the protocol when compared to the control group. Finally, hypothesis four is that we expect a decrease of depressive symptoms, measured through the Geriatric Depression Scale (GDS) [18], in the experimental group by the end of the protocol when compared to the control group. Further, we will assess whether these expected improvements in mental health and psychological well-being are maintained during a three-month follow-up period.

### Participants and inclusion criteria

The present study is a randomized controlled trial, which involves two groups of participants living in the ageing research and treatment center of Istituto Auxologico Italiano: the experimental group (EG) and the control group (CG). The total sample size is 40 participants, or 20 per group (sample size estimation details are reported in the following section).

**Figure 1:**
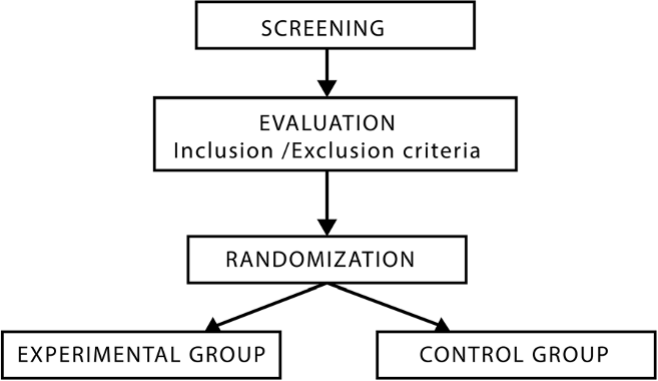
**CONSORT flow diagram.**

In order to be included in the study, individuals have to meet the following criteria: be 65 years of age or older; have an absence of major depressive symptoms, as indicated by a score lower than 17 on the GDS [18]; have an absence of cognitive impairment, as indicated by a score greater than 21 on the Mini Mental State Examination that corresponds to mild dementia [19]; and are not currently receiving any other psychological treatment for improving wellbeing. Both males and females will be included (Figure 1).

### Sample size calculation

The number of participants was calculated in order to have a sufficient size to prove the effect of training, reducing type two (false negative) statistical errors. The limit of 0.05 will be used for type one errors (false positive).

Sample size calculation used an assumption that participants in the experimental group would report improvements, based on outcome over time, and that these effects would differ from those achieved in the control group. As noted in the introductory section, previous evaluations of this kind of reminiscence intervention have produced moderate effect sizes [20, 21]. Accordingly, we estimated a sample size for a moderate treatment effect over time. We calculated sample size by using a repeated-measures analysis of variance across time (pre-post) with an α level set at 0.05 (two-tailed), statistical power level (1-β) of 0.80, and an estimate of correlation among repeated measures of 0.5. The calculation, based on GPower 3 (Heinrich-Heine-Universität, Düsseldorf, Germany) [22], led us to estimate minimum sample size of 40 participants (or 20 per group).

### Questionnaires

#### Screening

The intake session will use the following questionnaires: the Mini Mental State Examination [23] to assess the cognitive level and the Geriatric Depression Scale [18] to evaluate the depressive symptoms.

#### Outcome measures

For the assessment of group reminiscence outcome, we will administer several questionnaires at baseline, post-intervention, and three months post-intervention. The Life Satisfaction Index ([16]; Italian validation [24]) is a questionnaire used to assess satisfaction of life that includes 20 items with three possible answers of ‘yes’, ‘no’, or ‘do not know’. The Life Satisfaction Index covers feelings of wellbeing among older people to identify ‘successful’ aging; it includes components like joy, resilience, comparison between desired and achieved goals, positive view of the self, and mood tone. It is assessed by evaluating the individual’s way of facing daily activities, finding life meaningful, reporting a feeling of success, and a positive self-image. The Rosenberg Self-Esteem Scale ([15]; Italian validation [25]) is a questionnaire with 10 items in which the respondents will be asked to reflect on their current feelings. Self-esteem is measured through four-point Likert scales from ‘strongly disagree’ to ‘strongly agree’. The scale score ranges from 0 to 30, with a higher score indicating higher self-esteem. Scores between 15 and 25 are within the normal range and scores below 15 indicate low self-esteem.

The WHOQOL-Old [17] questionnaire measures the perceived quality of life, with 24 items assessing various aspects of the quality of life, using five-point Likert scales. The scale is split into six subscales which explore different aspects: sensory abilities, autonomy, past, present and future activities, social participation, death and dying, and intimacy. The Geriatric Depression Scale ([18]; Italian adaptation [26]) evaluates the presence of depressive symptoms among older adults. It is composed of 30 items with possible answers of ‘Yes’ or ‘No’. One point is given to each answer based upon a scoring grid, and then the cumulative score is calculated. The simplicity of this scale is appropriate for older adults. The Reminiscence Function Scale [27] is only for the experimental condition. This questionnaire includes 43 items investigating the main functions and purposes of the reminiscing activity over the life course using six-point Likert scales. Seven factors have been identified: boredom reduction, death preparation, identity and problem-solving, conversation, intimacy maintenance, bitterness revival, and teach and inform.

### Data collection

Therapists (clinical psychologists) will record psychological data from each individual for the entire study. Security and privacy issues will be addressed according to the respective situation and the consent of the participant. Questionnaires will be collected at the end of all the scheduled sessions and at the three-month follow-up.

### Trial design

In order to study the efficacy of the group reminiscence intervention, a between-subjects design will be used, with two experimental conditions and repeated measures across baseline, post-intervention, and three months post-intervention. Specifically, we will compare the following conditions. Experimental group: individuals will participate in group reminiscence meetings dealing with specific phases of their lives; they will share their memories and listen to the ones related by the other participants. Control group: individuals will participate in group conversations about current events.

Informed consent will be obtained from each participant. Psychometric outcomes will serve as quantitative dependent variables.

### Randomization

The present study is a randomized controlled trial. Participants will be randomly assigned to one of the two groups of the trial, balanced on gender and pre-test scores.

### Protocol

The intervention will last six weeks. Each week participants in both the experimental group and the control group will attend a group setting; in the experimental condition, participants will share their memories with peers, and in the control condition, participants will talk about current news.

### Setting

The guidelines reported in group reminiscence literature [28] will be followed to structure our reminiscence intervention. Each meeting will include six participants and last 60 minutes. Two clinical psychologists will lead the sessions. The physical setting will be in the nursing home; a comfortable room will be provided, accessible to wheel-chairs, and participants will sit in a circle. The facilitating psychologists will encourage involvement of each participant, in order to stimulate the discussion or debate in a positive atmosphere. Personal criticism, moral judgments, and political statements will be averted. The detailed protocol is described below, for both the experimental and the control group.

#### Experimental group

Participants will be assured that all the memories shared during the sessions will be confidential and will not be disclosed outside of the group. Every session will incorporate a theme, recalling specific autobiographic experiences in a chronological order. From the first to the sixth meeting, the topics will be best memory, childhood, school years, war, job, and holiday. Participants will be encouraged to bring photos or objects related to past themes. At the end of each meeting, the theme of the following session will be disclosed. The researchers can also bring materials as cues for reminiscence.

#### Control group

Every meeting will offer topics for discussion taken from newspaper and newscasts. Personal opinions will be promoted, and links with the daily life of participants will be welcome (everyday activities and personal preferences). A group discussion about these various themes will take place in every meeting from the first to the sixth. The main goal is to stimulate social interaction and communication, without discussing either personal events from the past or private memories. Final individual meetings will be scheduled for participants in both groups in order to administer the questionnaire and collect feedback from the users.

### Trial analysis

Data analyses will use IBM SPSS Statistics software (IBM, Armonk, USA). Descriptive methods will be used to demonstrate the consistency of the two groups, to describe participants’ characteristics, and to report levels of participation and dropout. Analysis of variance will compare baseline characteristics of the two groups involved in the study, and determine overall significance of improvement across the outcome measures.

### Ethics approval

This trial (project ID: 03A901) was approved by the Ethics Committee in the Istituto Auxologico Italiano, Milano (Italy) on 11 September 2012.

## Discussion

As the age of the population continues to rise, an increasing number of older individuals will enter a nursing home for professional care assistance. Defining effective strategies to help the elderly in adapting to the long-term care environment and maintaining their mental health will be a major challenge for researchers and practitioners. It is important for the nursing home staff to understand the many losses that residents face as they enter the facility, and the possible reactions they may have because of this life-changing event. Living in a nursing home may lead to depression, loss of self-efficacy, and loss of trust in personal skills and resources. A group reminiscence program, with a specific path guiding older people to re-evaluate their past life events, has the potential to strengthen the personal value of the institutionalized individual and foster their sense of identity. Listening to others, responding to them, and feeling close to life events of other people could provide a benefit in the quality of life; the elderly would find a safe place where they can experience positive interactions. Effectiveness of reminiscence therapy has received experimental support in several studies [6], yet the assessment of the specific benefits of group reminiscence as a ‘standard’ socializing activity in nursing homes is still an under-investigated issue [29]. It is expected that the outcomes of this trial will contribute to our knowledge about the process of group reminiscence, evaluate its effectiveness in improving psychological wellbeing of institutionalized individuals, and identify the best conditions for optimizing this approach.

## Trial status

Patient recruitment was ongoing at the time of manuscript submission. Recruitment began October 2012. Data collection will continue at least until June 2014.

## Abbreviations

CG: Control Group
EG: Experimental Group
GDS: Geriatric Depression Scale.
